# Facilitators and barriers to stakeholder engagement in advance care planning for older adults in community settings: a hybrid systematic review protocol

**DOI:** 10.12688/hrbopenres.13082.1

**Published:** 2020-06-23

**Authors:** Monika Pilch, Victoria Lunt, Peter May, David Mockler, Stephen Thomas, Frank Doyle

**Affiliations:** 1 Centre for Health Policy and Management, School of Medicine, Trinity College Dublin, University of Dublin, Dublin, D2, Ireland; 2 Beaumont Hospital and St Luke's Radiation Oncology Centre at Beaumont Hospital, Beaumont Hospital, Beaumont, Dublin, D9, Ireland; 3 The Irish Longitudinal study on Ageing (TILDA), Trinity College Dublin, Dublin, D2, Ireland; 4 The Library of Trinity College Dublin, Trinity College Dublin, University of Dublin, Dublin, D2, Ireland; 5 Division of Population Health Sciences, Royal College of Surgeons in Ireland, Dublin, D2, Ireland

**Keywords:** Advance care planning, community, behaviour change, systematic review

## Abstract

**Background: **Poor stakeholder engagement in advance care planning (ACP) poses national and international challenges, preventing maximisation of its potential benefits. Conceptualisation of advance care planning as a health behaviour highlights the need to design innovative, evidence-based strategies that will facilitate meaningful end-of-life care decision-making.

**Aim: **To review systematically and synthesise quantitative and qualitative evidence on barriers and facilitators to stakeholders` engagement in ACP for older adults (≥ 50 years old) in a community setting.

**Methods:** A hybrid systematic review will be conducted, identifying studies for consideration in two phases. First, databases will be searched from inception to identify relevant prior systematic reviews, and assess all studies included in those reviews against eligibility criteria (Phase 1). Second, databases will be searched systematically for individual studies falling outside the timeframe of those reviews (Phase 2). A modified SPIDER framework informed eligibility criteria. A study will be considered if it (a) included relevant adult stakeholders; (b) explored engagement in ACP among older adults (≥50 years old); (c) employed any type of design; (d) identified enablers and/or barriers to events specified in the Organising Framework of ACP Outcomes; (e) used either quantitative, qualitative, or mixed methods methodology; and (f) evaluated phenomena of interest in a community setting (e.g., primary care or community healthcare centres). Screening, selection, bias assessment, and data extraction will be completed independently by two reviewers. Integrated methodologies will be employed and quantitative and qualitative data will be combined into a single mixed method synthesis. The Behaviour Change Wheel will be used as an overarching analytical framework and to facilitate interpretation of findings. The Joanna Briggs Institute (JBI) Reviewers` Manual and PRISMA-P guidelines have been used to inform this protocol development.

**Registration: **This protocol has been submitted for registration on PROSPERO and is awaiting review.

## Introduction

People living and dying with serious illness are a societal and policy priority worldwide ( Centeno & Arias-Casais, 2019). As prognosis worsens and the end-of-life phase approaches, many people with terminal illness continue to receive aggressive, high-intensity treatment; e.g., chemotherapy in advanced cancer, mechanical ventilation in respiratory failure, and dialysis in chronic kidney disease. These treatments are often instigated and continued without checking patient preferences ( Kelley *et al.*, 2010), and as a result of poor physician understanding of those preferences ( Downey *et al.*, 2013). This may result in failure to address quality of life domains, such as pain and symptom management, or psychosocial and spiritual supports; often with limited survival benefit ( Earle *et al.*, 2008). They may result in longer hospital stays at a time when many people prefer to be at home or in hospice, and this time in hospital reduces remaining quality of life and time with loved ones ( Davison, 2010; Gomes *et al.*, 2012).

Advance care planning (ACP) provides mechanisms through which individual preferences can be meaningfully communicated and supported. A broad definition of ACP indicates that communication and decision-making processes regarding future healthcare wishes should include identification of patients’ goals of care, values, preferences, and priorities; and not be limited to mere completion of advance care directives. It also emphasises the importance of involving all relevant stakeholders and all domains of care; including biological, psychological, social, and spiritual ( Rietjens *et al.*, 2017). Although older people who engaged in ACP are less likely to be admitted to intensive care units (ICUs), acute care settings (e.g., ICUs or emergency departments) are not ideal contexts for the initiation of end-of-life conversations or decision-making ( Khandelwal *et al.*, 2015). Similarly, individuals presenting to an emergency department from residential aged care settings are more likely to have an advance care plan than community dwellers ( Street *et al.*, 2014). Therefore, opportunities to engage individuals in ACP at an early stage and facilitate ongoing conversations regarding future healthcare needs in a community, non-residential setting (e.g., primary care or community healthcare centres) needs to be explored to bridge this gap.

ACP is associated with increased quality of life ( Garrido *et al.*, 2015); a reduction in unwanted admissions to hospitals; care consistent with patients` goals ( Jimenez *et al.*, 2018); improved quality of end-of-life care, increased use of hospice services and reduced hospital deaths ( Bischoff *et al.*, 2013); compliance with patients` end-of-life wishes ( Brinkman-Stoppelenburg *et al.*, 2014; Silveira *et al.*, 2010); decreased caregiver burden ( Balein, 2009; Earle *et al.*, 2008); better bereavement processes and more positive psychological outcomes for family members ( Wright *et al.*, 2008); reduced health care spending ( Gidwani-Marszowski *et al.*, 2019; Zhang *et al.*, 2009); and cost-effective strategies for facilitating patients` choice ( Nguyen *et al.*, 2017). However, low engagement in ACP poses national and international challenges, preventing maximisation of these potential benefits.

Community-based interventions have the potential to overcome many of the barriers to ACP, to include the introduction of a more person-cantered, holistic, inclusive, population-based, and integrated care approach. The biggest challenge to ACP implementation is the identification of the right time to initiate these sensitive end-of-life conversations. Diagnosis is often associated with increased psychological distress, which might impact patients` readiness, mental ability to comprehend novel medical concepts, and decision-making capacity ( Pinquart & Duberstein, 2010). Although clinical guidelines specify that ACP should commence when there is evidence of a life-limiting advanced progressive illness ( Clayton *et al.*, 2007), research findings and clinical practice suggest that this might be too late ( Jimenez *et al.*, 2018). The preparation for end-of-life conversations is a process that could begin early in the life course, e.g., when individuals reach their 50 ^th^ year of life, and irrespective of health status ( Howard *et al.*, 2018). As this age group is at an increased risk of presenting with a chronic illness ( Mullaney *et al.*, 2016), end-of-life issues are likely to be personally relevant to them ( Simon *et al.*, 2015). As readiness to ACP engagement is linked to the concept of preparedness, the interventions introduced at an *early* stage, in a community, could prepare individuals for patient-healthcare practitioner end-of-life conversations in specialised palliative care settings, and take place when individuals are ageing but still healthy ( Lewis *et al.*, 2016).

Irrespective of supportive legal frameworks and relevant policies ( Kelly, 2017), patient engagement in ACP is poor ( Brinkman-Stoppelenburg *et al.*, 2014; Jimenez *et al.*, 2018). Effectiveness studies suggest that an *early* and *phased process* of preparation for the last stage of life, incorporating: 1) longitudinal and repeated ACP discussions ( Prendergast, 2001); 2) customised to patients` needs, circumstances, and readiness ( Fried *et al.*, 2009; Simon *et al.*, 2015); 3) involving digital decision aids ( Austin *et al.*, 2015; Ostherr *et al.*, 2016); 4) targeting multiple stakeholders; and 5) taking context into account ( Jimenez *et al.*, 2018) would increase *informed* and *meaningful* participation in ACP, as well as optimise end-of-life outcomes.

There is a growing recognition of engaging in ACP as a *health behaviour* ( Fried *et al.*, 2009; Fried *et al.*, 2010; Sudore *et al.*, 2008). This behavioural conceptualisation allows a view of ACP as subject to health behaviour change interventions ( Fried *et al.*, 2012; Fried *et al.*, 2016; Fried *et al.*, 2018; Sudore *et al.*, 2017; Van Scoy *et al.*, 2017). To date, most approaches to behaviour change have focused on individuals, and although this level is crucial when highly sensitive ACP conversations are considered, approaches that ignore contextual influences are open to criticism ( Lin *et al.*, 2019). The Organising Framework of ACP Outcomes suggests that readiness to ACP engagement is a complex construct, which needs to be systematically assessed with regards to all relevant stakeholders. Therefore, a comprehensive systematic synthesis of findings, across all aspects of the framework, is essential to informing efforts in behaviour change. This proposed conceptualisation of ACP supports the use of the Behaviour Change Wheel (BCW) framework ( Michie *et al.*, 2011), which is an encapsulating model, and increasingly employed in ACP empirical investigations (e.g., Biondo *et al.* 2019; Peck *et al.*, 2018; Tam-Tham *et al.*, 2016).

Given the need to increase meaningful ACP engagement, the design of innovative interventions; targeting complex interactions between facilitators, barriers, and stakeholders, is required. There is a consensus that the development of ACP interventions should be based on a careful and critical synthesis of available evidence and the understanding of barriers and enablers to engagement ( Risk *et al.*, 2019). However, existing systematic reviews tend to focus on acute and specialised contexts ( Gilissen *et al.*, 2019), one group of stakeholders ( De Vleminck *et al.*, 2013), and/or specific clinical conditions ( Tilburgs *et al.*, 2018; Van Der Steen *et al.*, 2014). Although several studies have been conducted to explore factors associated with the uptake of ACP among older adults in community settings; and these have been synthesised in previous systematic reviews (e.g., De Vleminck *et al.*, 2013; Ramsaroop *et al.*, 2007; Risk *et al.*, 2019; Solis *et al.*, 2018), no study has synthesised available evidence to identify patterns across older adults, irrespective of their health status or condition, while taking stakeholder perspective and level of influence into account. A synthesis utilising the BCW framework is necessary to identify influences within the contextual layers of health behaviour among older adults living in a community, while highlighting individual, interpersonal, provider, and system levels of influence.

### Objectives and review questions

The overarching aim of the study will be to systematically review and synthesise quantitative and qualitative evidence on barriers and facilitators to stakeholders` engagement in ACP for older adults (≥ 50 years old) in a community setting.

The synthesis will be guided by the following review questions (RQs):

- RQ1: What individual-level, service-level, and system-level factors facilitate and/or impede stakeholders` ACP engagement for older adults in a community setting?
- RQ2: When categorised into the COM-B (‘capability’, ‘opportunity’, ‘motivation’ dimensions of behaviour) model, which of the identified barriers and facilitators are grouped into the categories of (a) Capability, (b) Motivation, and (c) Opportunity?

## Methods

### Design

As several systematic reviews have been published in this area, a hybrid systematic review will be completed, adopting the methodology of Doyle *et al.* (2019). In a hybrid review approach, potentially relevant studies are searched for in two phases ( Doyle *et al.*, 2019). First, a systematic search for systematic reviews will be completed and eligible primary studies included in those reviews will be identified and extracted (Phase 1). Second, this process will be supplemented with an updated systematic review of more recently published individual studies (Phase 2). When available, relevant data will be extracted from the eligible systematic reviews. If not available, two reviewers will independently extract data from the primary studies.

While no prior systematic review has examined the specific research questions, multiple prior reviews have addressed questions that partially overlap. The advantage of the hybrid method is, therefore, that it leverages the efforts of multiple prior studies and minimises duplication of effort with those same teams. Derived results will nevertheless represent a distinctive contribution to knowledge in specifically addressing, for the first time, the barriers and facilitators for ACP for older people (≥50 years old) in a community setting; which is important for the reasons detailed elsewhere in this protocol.

A mixed-method approach will be used and quantitative, qualitative, and mixed-methods reviews will be included. The BCW will be used as an overarching analytical framework ( Michie *et al.*, 2011). The Joanna Briggs Institute (JBI) Reviewers` Manual ( Lizarondo *et al.*, 2019) and PRISMA-P guidelines ( Moher *et al.*, 2015) have been used to inform this protocol development (see *Reporting guidelines*, Pilch, 2020). This protocol has been submitted for registration on PROSPERO and is awaiting review.

### Eligibility criteria

The modified SPIDER reporting framework ( Cooke *et al.*, 2012) informed eligibility criteria, which were specified for the two stages of the search: the search for systematic reviews (Phase 1) and the supplementary search for primary studies (Phase 2). A “setting” (“s”) eligibility criterion was added to incorporate a contextual variable (resulting in SPIDER”s” framework).

### SPIDERs reporting framework

A review or a primary study will be deemed eligible if it: (a) included adult stakeholders (relevant older adults≥ 50 years old, their significant others, healthcare professionals, non-medical peers, service managers, and/or policy makers) ( **Sample**); (b) explored engagement in ACP among older adults (≥ 50 years old) ( **Phenomena of Interest**); (c) employed any type of design ( **Design**); (d) identified enablers and/or barriers to events specified in the Organising Framework of ACP Outcomes ( Sudore *et al.*, 2018) ( **Evaluation**); (e) used either quantitative, qualitative, or mixed methods methodology ( **Research Type**); and (f) evaluated phenomena of interest (or included primary studies that aimed to do so) in a community setting ( **Setting)**. Only reviews and primary studies reported in English and published in peer-reviewed journals will be considered for inclusion. The necessary differences in the approach taken when identifying systematic reviews (Phase 1) or the supplementary primary studies (Phase 2), are presented in Table 1. The specification of the inclusion and exclusion criteria is presented below.

**Table 1.** Inclusion and exclusion criteria.

Search for systematic reviews (Phase 1)	Supplementary search for primary studies (Phase 2)
**Stakeholder:** Relevant older adults, their significant others, healthcare professionals, non-medical peers, service managers, and/or policy makers. • **Population of interest:** those in a community setting and >50 years old.	**Stakeholder:** Relevant older adults, their significant others, healthcare professionals, non-medical peers, service managers, and/or policy makers. • **Population of interest:** those in the community setting and >50 years old.
**Events or actions** associated with the stakeholders’ engagement, uptake, utilisation, implementation, and/or initiation of ACP (e.g., communication, decision making, documentation completion, etc.), as informed by the Organising Framework of ACP Outcomes ( Sudore *et al.*, 2018).	**Events or actions** associated with the stakeholders’ engagement, uptake, utilisation, implementation, and/or initiation of ACP (e.g., communication, decision making, documentation completion, etc.), as informed by the Organising Framework of ACP Outcomes ( Sudore *et al.*, 2018).
**Systematic reviews** that employed all types of review methodology. **Exclusion:** protocols, scoping reviews, narrative reviews, overviews of reviews, editorials, comments, and expert opinion.	**Empirical studies** • Descriptive studies as well as experimental and observational study designs that explored the link between predictor, mediator, and/or moderator variables and relevant ACP events and outcomes. • All qualitative approaches and designs.
**Facilitators and/or barriers** to events or actions specified in the Organising Framework of ACP Outcomes; and identified at the level of an individual, a service, and/or a system. • **A barrier**: any factor that has been identified as an obstacle, impediment, deterrent, hindrance, or difficulty in a stakeholder`s engagement, uptake, utilisation, implementation, and or initiation in ACP activities/events. • **A facilitator:** any enabler or factor that has been shown to increase the chances of a stakeholder`s engagement, uptake, utilisation, implementation, and or initiation in ACP activities/events.	**Facilitators and/or barriers** to events or actions specified in the Organising Framework of ACP Outcomes; and identified at the level of an individual, a service, and/or a system. • **A barrier**: any factor that has been identified as an obstacle, impediment, deterrent, hindrance, or difficulty in a stakeholder`s engagement, uptake, utilisation, implementation, and or initiation in ACP activities/events. • **A facilitator:** any enabler or factor that has been shown to increase the chances of a stakeholder`s engagement, uptake, utilisation, implementation, and or initiation in ACP activities/ events.
All types of quantitative, qualitative, and mixed-methods review methodologies will be included in the review.	Quantitative, qualitative, and mixed-method approaches employed in the primary studies will be considered for inclusion.
Reviews that included studies that meet the inclusion criteria for the primary studies. If evidence from multiple settings were included, a review will be included only if data relating to community settings can be extracted separately. **Exclusion:** Studies conducted in the hospital (e.g., ICU or an emergency department), ambulatory setting, outpatient or hospital clinics, and/or long term care facilities (e.g., residential care, to include a nursing home or a hospice) will be excluded.	Studies conducted in, or related to, all kind of community-based centres and/or primary care. Eligibility determined by the setting of receiving care. Studies from both rural and urban settings will be included. **Exclusion:** Studies conducted in the hospital (e.g., ICU or an emergency department), ambulatory setting, outpatient or hospital clinics, and/or long term care facilities (e.g., residential care, to include a nursing home or a hospice) will be excluded.

ACP, advance care planning; ICU, intensive care unit.

***Sample.*** In the context of this review, the eligible population includes adult stakeholders who have the potential to influence ACP engagement among older adults in a community setting. They include older adults themselves, their significant others, healthcare professionals, non-medical peers, service managers, and/or policy makers. A study will be deemed eligible if it collected data related to the population of interest; adults in community settings, aged ≥50 years old. This population has been chosen as this age group is at increased risk of presenting with a chronic illness, therefore, end-of-life issues are likely more relevant to them ( Mullaney *et al.*, 2016). There will be no limits on the health status and/or type of a condition or diagnosis as capturing the diversity of perspectives of various groups and the identification of common patterns across diagnoses, as well as healthy participants, is an ultimate goal of this review.

***Phenomena of interest.*** The focus of this review will be on engagement (or not) in ACP among older adults in a community setting. The conceptualisation of the phenomena of interest was informed by the Organising Framework of ACP Outcomes ( Sudore *et al.*, 2018) and involves events or actions associated with the stakeholders’ engagement, uptake, utilisation, implementation, and/or initiation of ACP. Therefore, in the context of this review, the relevant events and/or outcomes include, but are not limited to: (a) documentation of values, goals, and/or preferences (e.g., completion of the living will and/or advance directives); (b) the choice and/or documentation of a decision-maker; (c) communications about the goals of care, to include quality vs quantity of life (with family and/or health-care providers); and (d) communications about life-sustaining treatments (with family and/or health-care providers).

***Design.*** Empirical studies that employed all types of methodology will be included in this review. In the first stage of the search (Phase 1), only systematic reviews will be selected and protocols, scoping reviews, narrative reviews, overviews of reviews, editorials, comments, and expert opinion will be excluded. When selecting primary studies, descriptive, experimental, and observational study designs that explored links between variables identified as facilitators or barriers and relevant ACP events/outcomes will be considered for the quantitative component of the review. An inclusive approach will be taken and the qualitative component of the review will not be limited by a specific research design.

***Evaluation.*** Facilitators and/or barriers to events specified in the Organising Framework of ACP Outcomes ( Sudore *et al.*, 2018) will be the core evaluation in the context of this study. Therefore, reviews and/or primary studies exploring individual, service, and system level factors that facilitated or impeded stakeholders’ engagement, uptake, utilisation, implementation, and or initiation in ACP activities/events (communication, decision making, documentation completion, etc.) will be included. For the purposes of this review, a barrier has been broadly defined as any factor that has been identified as an obstacle, impediment, deterrent, hindrance, or difficulty. A facilitator is defined as any kind of an enabler or factor that has been shown to increase the chances of ACP engagement. Following the model proposed by Sudore *et al.* (2018), studies that explored relevant factors in relation to individuals (older adults, significant others, healthcare professional, service managers, and/or policy makers), communities (public health, community incentives, legal support, policy and media), and healthcare system (documentation, training, facilitators, and palliative care) will be included.

When selecting primary studies, those that explored predictors, moderators, and/or mediators of ACP engagement will be included in the quantitative component of the review. The quantitative component will also consider studies that identified factors (e.g., active ingredients) contributing to the effectiveness of interventions, tools, and/or strategies designed to increase engagement in ACP activities or events. The qualitative component of the review will consider studies that explored perspectives, views, opinions, and/or experiences of relevant stakeholders in relation to the phenomena of interest.

***Research type.*** All types of quantitative, qualitative, and mixed-methods review methodologies will be included in the first stage of the review (Phase 1). Quantitative, qualitative, and mixed-method approaches employed in the primary studies will be considered for inclusion (Phase 1 & 2).

***Setting.*** A review will be eligible only if it included studies conducted in community, non-residential settings. In the selection of primary studies, the setting of receiving care will determine whether a study is eligible. Therefore, a study will be included if it recruited patients from all kind of community-based centres and/or primary care. Studies that took place in the hospital (e.g., ICU or an emergency department), outpatient or ambulatory settings, hospital clinics, and/or long term care facilities (e.g., residential care, to include a nursing home or a hospice) will be excluded. This decision has been made as the aim is to explore opportunities to facilitate the initiation of end-of-life conversations at an early stage, when individuals are relatively well. Although it is acknowledged that hospital-based services may have this function for some individuals, and in certain contexts, the aim of this synthesis is to enhance an understanding of patterns of ACP engagement outside of long-term and acute care environments. If evidence from multiple settings were synthesised in the primary review, a review will be included only if data relating to relevant settings can be extracted separately from non-relevant settings.

### Search strategy

As a hybrid systematic review will be conducted ( Doyle *et al.*, 2019), a two-stage search strategy has been developed in collaboration with a medical librarian (DM). A comprehensive search for systematic reviews will be conducted in each database from its inception (Phase 1). The eligible reviews will be used to identify and extract relevant primary studies. This data will be supplemented by an updated search for more recent studies; falling outside the timeframe of those reviews (Phase 2). The search strategy will also include screening of relevant overviews of reviews (in Phase 1), “backward and forward” citation search (Phase 1 & 2), and reference list screening (Phase 1 & 2).

***Electronic searches.*** All searches will be completed by the first reviewer (MP). The search for systematic reviews (Phase 1) will include comprehensive searching of electronic databases from inception, and will include MEDLINE (Ovid), EMBASE, PsycINFO, the Cochrane Library (CENTRAL), CINAHL, and Epistemonikos. A sample search strategy for EMBASE in Phase 1 of the search is included in Appendix B (see *Extended data*, ( Pilch, 2020)).

It is estimated that the time limit for the supplementary identification of primary studies (Phase 2) will be the last three years; however, it will be determined and specified after completing Phase 1 of the search. The supplementary search will include comprehensive searching of the same databases, with the addition of the Web of Science. Epistemonikos will not be searched in Phase 2 as it is likely to identify systematic reviews and primary studies already included in the identified reviews ( El-Khayat, 2017). A sample search strategy for EMBASE in Phase 2 of the search is included in Appendix C (see *Extended data*, ( Pilch, 2020). The search strategy will be adapted to each database interface.

The search terms used are specified in Table 2. In searching for systematic reviews, terms relating to the phenomena of interest, design, evaluation, and setting will be utilised in the database searches, combined with ‘AND’. In the supplementary search for primary studies, the search terms relating to the design category will not be used.

**Table 2.** Search Terms.

	Search Terms
**Phenomena of** **Interest**	‘advance* care plan*’ OR ‘patient advance* care plan* OR ‘advance* health care plan*’ OR ‘advance* healthcare plan*’ OR ‘anticipat* care’ OR ‘anticipat* healthcare’ OR ‘anticipat* health care’ OR ‘advance* directive*’ OR ‘healthcare directive*’ OR ‘advance* care statement*’ OR ‘living will’ OR ‘resuscitation order*’ OR ‘do not resuscitate’ OR ‘DNR’ OR ‘DNAR’ OR ‘ADRT’ OR ‘right to die’ OR ‘right-to-die’ OR ‘do not hospitali?e’ OR ‘prefer* place* of care’ OR ‘prefer* place* of death’ OR ‘end of life prefer*’ OR ‘end-of-life prefer*’OR ‘EOL prefer*’ OR ‘end of life plan’ OR ‘end-of-life plan’ OR ‘EOL plan’ OR ‘end of life choice’ OR ‘end-of-life choice’ OR ‘EOL choice’ OR ‘end of life decision*’ OR ‘end-of-life decision*’ OR ‘EOL decision*’ OR ‘end of life communication*’ OR ‘end-of-life communication*’ OR ‘EOL communication*’ OR ‘end of life conversation*’ OR ‘end-of-life conversation*’ OR ‘EOL conversation*’ OR ‘end of life care goal*’ OR ‘end-of-life care goal*’ OR ‘EOL care goal*’ OR ‘end of life document*’ OR ‘end-of-life document*’ OR ‘EOL document*’ OR ‘end of life directive*’ OR ‘end-of-life directive*’ OR ‘EOL directive*’ OR ‘disease-specific advance* plan*’ OR ‘chronic illness advance* plan*’ OR ‘progressive illness advance* plan*’ OR ‘patient advance* plan*’ OR ‘patient advance* directive*’ OR ‘patient advance* statement*’ OR ‘patient advance* decision*’ OR ‘prefer* place* of care’ OR ‘prefer* place* of death’ OR ‘patient care plan*’ OR ‘anticipatory care’ OR ‘anticipatory plan’ OR ‘health care prox*’ OR ‘healthcare prox*’ OR ‘power of attorney’ OR ‘surrogate decision-maker’ OR ‘surrogate decision maker’ OR ‘decision making representative*’ OR ‘decision- making representative*’
**Design**	‘review’ OR ‘systematic review’ OR ‘synthesis’ OR ‘meta-synthesis’ OR ‘meta-analysis’
**Evaluation**	barrier* OR facilitator* OR impediment* OR obstacle* OR deterrent* OR difficult* OR enabler* OR promot* OR benefit* OR burden*
**Setting**	‘community care’ OR ‘community health care’ OR ‘community healthcare’ OR ‘primary care’ OR ‘primary health care’ OR ‘primary healthcare’ OR ‘‘general practice’ OR ‘general practitioner’ OR ‘family practice’ OR ‘community-dwelling person’ OR ‘community-dweller’ OR ‘home care’

### Screening and selection of primary studies

**Figure 1.**
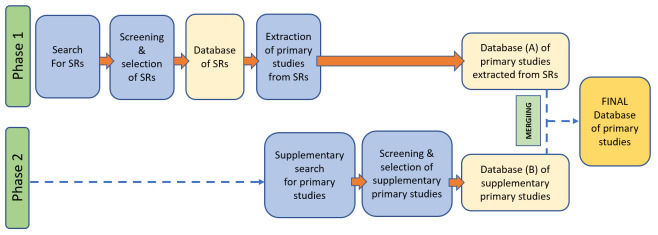
A two-stage process of primary studies identification. SRs, systematic reviews.

The two-stage process of primary studies identification is illustrated in Figure 1. Upon completion of the first stage of the search (Phase 1), all references will be downloaded into Endnote. Duplicates will be removed using the automatic function of the software. Then, the references will be exported to Covidence. Titles and abstracts will be independently screened by the first and second reviewer (MP & VL) and those clearly not relevant to the review will be eliminated. Full-text copies of reports will be retrieved for all papers passing title/abstract review. Full texts will be screened by MP and VL and those clearly not relevant to the review will be eliminated. Conflicts will be resolved through discussion and consensus with other reviewers (PM, FD, or ST). A database of eligible *systematic reviews* will be created at the end of this process.

The eligible primary studies will be independently extracted from the selected systematic reviews by MP and VL. Eligibility of primary studies will be determined by screening the summary tables included in the systematic reviews (if available) or by reviewing the full report (if summary tables are not available or do not include relevant information). A database of the eligible primary studies will be created at this stage (Database A in Figure 1).

Upon completion of the second stage of the search (Phase 2), all references will be downloaded into Endnote and duplicates removed. The systematic processes described above will be applied to the supplementary search and will include: screening of titles and abstracts and elimination of non-relevant studies (MP & VL); retrieval of full-text copies of reports for all papers passing title/abstract review (MP & VL); screening of the full-texts and elimination of those clearly not relevant to the review (MP & VL). Conflicts will be resolved through discussion and consensus with other reviewers (PM, FD, or ST). A database of primary studies identified during the supplementary search will be created at the end of this process (Database B in Figure 1). Both databases (Database A & Database B) will be merged.

Multiple reports of the same study, if identified, will be treated as a single study. The details of both screening and selection processes (one for systematic reviews and one for primary studies), with the indication of included and excluded studies at each stage, will be documented and demonstrated on the PRISMA flow diagrams. Reasons for rejection at the full-text stage will be recorded.

### Assessment of risk of bias in included studies

As the primary studies will be extracted from the eligible systematic reviews, the quality assessment of those studies will also be extracted (if available). If not available, the risk of bias assessment will be completed by MP and VL. The Crowe Critical Appraisal Tool (CCAT); a validated general critical appraisal instrument that can be used across a wide range of research designs, will be used ( Crowe *et al.*, 2012). A similar procedure will be followed if reviews disagree on the assessment of bias of the included primary studies. The quality of newly identified primary studies (not included in the eligible systematic reviews) will also be assessed with the CCAT tool. Other reviewers (PM, FD, or ST) will be involved, if necessary. The appraisal checklist will be computerised and the electronic version will be used to facilitate critical assessment process. The tool will be used to identify bias but not to exclude studies.

### Data extraction and management

Two data extraction forms will be designed to ensure a focused and systematic approach to data collection. The form for the extraction of data from systematic reviews will be informed by the JBM instrument for the conduct of umbrella reviews ( Aromataris *et al.*, 2017). The JBM Mixed Methods data extraction form will be used to develop a data extraction form for individual studies ( Lizarondo *et al.*, 2019). Each form will be piloted on a sample of three studies (reviews or individual studies, respectively) and adapted, if necessary. When finalised, the forms will be computerised and the electronic versions will be used to facilitate data extraction processes.

The following information will be extracted from the reviews: (a) review details (authors, year of publication, journal, review type, overarching conceptual frameworks, aims and objectives); (b) search details (sources searched, range of included studies, number of studies included in the review, type of included studies); (c) summary findings, including tabulated individual study details; (d) details of quality appraisal (appraisal tool used and appraisal rating); (e) details of analysis and analytical approach (analytical framework and method of analysis); (f) results/findings and authors conclusions.

The following information will be extracted from the individual studies: (a) study details (authors, year of publication, journal, title, geographical area of the study, overarching conceptual frameworks); (b) methodology (study design, aims and objectives, context/settings, participant characteristics, description of phenomena of interest; (c) details of analysis/ analytical approach; (d) details of evaluation (outcomes or findings of significance to the review objectives, themes or subthemes and associated illustration), (e) authors’ conclusions. If available, data relevant to the review questions will be independently extracted from the eligible systematic reviews by the first and the second reviewer (MP & VL). If not available, a standard procedure will be followed to extract data from the eligible primary studies. The same process of data extraction will be repeated for the primary studies identified thorough the supplementary search (Phase 2). Conflicts will be resolved through discussion and consensus. Other reviewers (PM, FD, or ST) will be involved if there is a disagreement. To obtain information on missing data, authors will be contacted.

### Analytical approach and integration of findings

**Figure 2.**
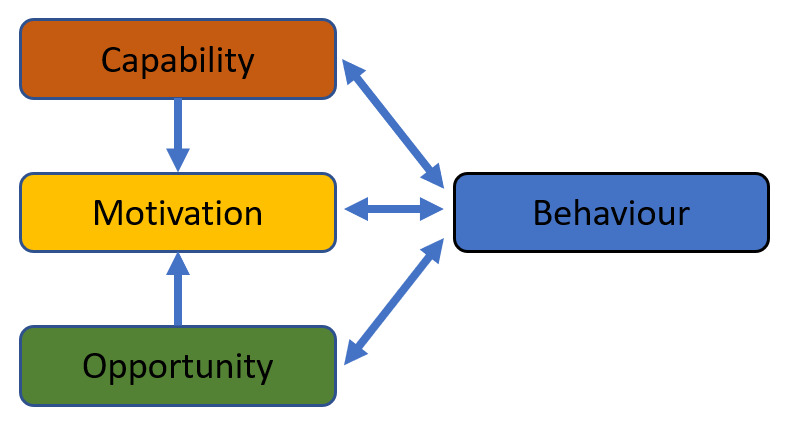
The COM-B system (adapted from: Michie *et al.*, 2011, under a CC BY 2.0 license).

Integrated methodologies will be employed and quantitative and qualitative data will be combined into a single mixed research synthesis ( Sandelowski *et al.*, 2006). Data will be integrated by translating qualitative data into numerical format. This converted data will be presented along with quantitative data in a summary table ( Lizarondo *et al.*, 2019). Then, following the similar approach taken elsewhere ( Sharpe *et al.*, 2018), the findings will be mapped across the COM-B categories (see Figure 2). Specifically, MP and VL will record the frequency of reported facilitators and barriers and group the findings into the relevant categories of the COM-B model. Then, the BCW ( Michie *et al.*, 2011) will be utilised as a broader analytical framework to differentiate individual-level, service-level, and system-level factors that facilitate and/or impede stakeholders` engagement in ACP. Other reviewers (PM, FD, & ST) will be asked to provide a critical evaluation of the analytical process and the findings.

### Dissemination

We will apply a peer-reviewed knowledge transfer and exchange model ( Payne *et al.*, 2019) and use multi-channel dissemination to reach different audiences and all key stakeholders. A systematic review article will be submitted for peer-reviewed publication.

### Study status

Preliminary searches have been completed.

## Discussion

Given the low engagement in ACP and the known challenges to introducing ACP in the last stage of illness trajectories, which usually occurs in acute healthcare settings, there is a need to explore salient factors facilitating ACP engagement in a community context. These settings are likely to offer space and time for ACP conversations, allowing their integration into standard care. This review will be the first to adopt a hybrid review methodology to present cumulative evidence on facilitators and barriers to ACP among older adults in a community setting, as perceived from the perspective of various stakeholders. By uniquely adopting the BCW framework, it will provide insights into different levels of influence (including individual, service-based, and systemic), and with the view of informing behaviour change strategies. This comprehensive approach will aim to enhance the understanding of modifiable and non-modifiable factors that may facilitate or impede early key stakeholders` engagement in ACP for older adults, irrespective of their health status.

The adoption of a specific methodology, combining a systematic search for systematic reviews with systematic supplementary searches for original studies, will allow a cumulative and time-effective approach. The strength of the hybrid approach is that it leverages the work undertaken in previous systematic reviews, e.g., by utilising the reported outcomes of comprehensive searches and selection processes ( Doyle *et al.*, 2019). Given the amount of existing evidence, this methodology will prevent the repetition of already completed and reported processes, facilitate more appropriate management of resources, and allow the application of sophisticated analytical frameworks to cumulative evidence.

The findings of this review may inform the development of an innovative and evidence-based interventions for ACP. The Medical Research Council Guidelines highlight the need to identify strong evidence base and model processes and outcomes to inform the development of complex healthcare interventions ( Craig *et al.*, 2019). As different settings may influence engagement in ACP ( Lewis *et al.*, 2016), the need to keep the implementation context in mind when designing and assessing interventions has been highlighted ( Edmondson *et al.*, 2001; Jimenez *et al.*, 2018). A careful and critical synthesis of available evidence, enhancing the understanding of barriers and enablers of ACP engagement in a community setting, can facilitate the development of relevant ACP interventions ( Risk *et al.*, 2019). While suggesting direction for further empirical investigations, the findings will also allow to derive conclusions and recommendations for clinical and policy decision making.

## Data availability

### Underlying data

No underlying data are associated with this article.

### Extended data

Open Science Framework: Facilitators and barriers to stakeholder engagement in Advance Care Planning for older adults in community settings: A hybrid systematic review protocol. https://doi.org/10.17605/OSF.IO/6TS34 ( Pilch, 2020).

### Reporting guidelines

Open Science Framework: PRISMA-P checklist for ‘Facilitators and barriers to stakeholder engagement in Advance Care Planning for older adults in community settings: A hybrid systematic review protocol.’ https://doi.org/10.17605/OSF.IO/6TS34 ( Pilch, 2020).

Data are available under the terms of the Creative Commons Attribution 4.0 International license (CC-BY 4.0).
