# Supplementary material for: Facilitators and barriers to stakeholder engagement in advance care planning for older adults in community settings: a hybrid systematic review protocol
Source: HRB Open Res. 2021 Jun 11;3:38. Originally published 2020 Jun 23. [Version 2] doi: 10.12688/hrbopenres.13082.2 (PMC8212429; doi:10.12688/hrbopenres.13082.2)
Supplement: Peer review history

# Peer review: Reviewer response for version 2

**DOI:** 10.21956/hrbopenres.14513.r29658

**Published:** 2021-06-17

**Authors:** Susan Fiona Lee

**Affiliations:** 1 Nursing and Midwifery, Nursing and Health Sciences, Monash University, Frankston, Vic, Australia

I have read the comments and revisions and find them to be thoughtful and having addressed my few concerns. The review adds to our understanding of the complexity of advance care planning.

Is the study design appropriate for the research question?

Partly

Is the rationale for, and objectives of, the study clearly described?

Yes

Are sufficient details of the methods provided to allow replication by others?

Partly

Are the datasets clearly presented in a useable and accessible format?

Not applicable

Reviewer Expertise:

Palliative care, end of life care, decision making, advance care planning.

I confirm that I have read this submission and believe that I have an appropriate level of expertise to confirm that it is of an acceptable scientific standard.

# Peer review: Reviewer response for version 1

**DOI:** 10.21956/hrbopenres.14182.r27813

**Published:** 2020-09-01

**Authors:** Susan Fiona Lee

**Affiliations:** 1 Nursing and Midwifery, Nursing and Health Sciences, Monash University, Frankston, Vic, Australia

Thank you for the opportunity to review this protocol for a novel systematic review. This mixed method review seeks to examine barriers and facilitators to stakeholder engagement in Advance Care Planning (ACP) in community settings. The context is well argued in relation to the need for earlier ACP. The review uses a novel hybrid approach by first searching for systematic reviews and following this, the subsequently published individual articles. Risk of bias assessments are to be conducted and rigour will be maintained through the use of independent selection of studies, risk of bias assessment and data extraction by two reviewers. A further novel approach is used in the analysis involving a mixed-method research synthesis, and findings will be mapped against the existing ACP outcomes framework. 

 The need for this review is clearly justified and it is a reasonably well thought out process. I recommend revisiting two areas. 

1. The hybrid approach does not include the common third search strategy of grey literature on the basis of a lack of peer review. I presume these studies will also not be extracted from the systematic reviews. As critique is part of the rigour of the review, studies that have not been peer reviewed, along with those that have been reviewed, undergo risk of bias assessment. I believe the exclusion of grey literature searching and studies should be reviewed. 
2. I found some confusion in the treatment of the systematic reviews discovered in phase 1. It appears from the search strategy that the systematic reviews are treated as a data base of eligible studies and the studies will be extracted and added to the phase 2 study list. However, in the data management section and analysis, the results of the reviews are extracted and used in the synthesis, as in an umbrella review. This must be clarified prior to data extraction.

As a final point, I did note that the search terms did not include any synonyms for surrogate. I recommend 'substitute*' and 'alternate' to ensure the scope of this group. There also appears a typographical error in the first paragraph of the Data extraction and management section where JBM is referred to. I believe it should be JBI.

 I wish you well with the review and look forward to reading the final outcomes which should have practical implications for the field.

Is the study design appropriate for the research question?

Partly

Is the rationale for, and objectives of, the study clearly described?

Yes

Are sufficient details of the methods provided to allow replication by others?

Partly

Are the datasets clearly presented in a useable and accessible format?

Not applicable

Reviewer Expertise:

Palliative care, end of life care, decision making, advance care planning.

I confirm that I have read this submission and believe that I have an appropriate level of expertise to confirm that it is of an acceptable scientific standard, however I have significant reservations, as outlined above.

# Response

**Published:** 2021-06-04

**Authors:** Monika Pilch

**Affiliations:** Trinity College Dublin, Ireland

We are grateful for the time taken by reviewers in providing a careful analysis of our protocol. We appreciate your positive comments and constructive suggestions to further improve our manuscript. We have revised the paper and  have made a point-by-point reply to your comments, as follows:

 1. Thank you for the opportunity to review this protocol for a novel systematic review. This mixed method review seeks to examine barriers and facilitators to stakeholder engagement in Advance Care Planning (ACP) in community settings. The context is well argued in relation to the need for earlier ACP. The review uses a novel hybrid approach by first searching for systematic reviews and following this, the subsequently published individual articles. Risk of bias assessments are to be conducted and rigour will be maintained through the use of independent selection of studies, risk of bias assessment and data extraction by two reviewers. A further novel approach is used in the analysis involving a mixed-method research synthesis, and findings will be mapped against the existing ACP outcomes framework. 

 The need for this review is clearly justified and it is a reasonably well thought out process

*Thank you for these positive comments.*

 2. The hybrid approach does not include the common third search strategy of grey literature on the basis of a lack of peer review. I presume these studies will also not be extracted from the systematic reviews. As critique is part of the rigour of the review, studies that have not been peer reviewed, along with those that have been reviewed, undergo risk of bias assessment. I believe the exclusion of grey literature searching and studies should be reviewed. 

*As stated in response to the first reviewer (comment 6), we decided to focus on peer-reviewed literature only. This has now been explicitly stated in the text (p. 11). As explained above, the inclusions of grey literature in this review would be beyond the scope of this PhD project. However, we will acknowledge this limitation in the discussion and will comment on the impact of this approach on findings.*

 3. I found some confusion in the treatment of the systematic reviews discovered in phase 1. It appears from the search strategy that the systematic reviews are treated as a data base of eligible studies and the studies will be extracted and added to the phase 2 study list. However, in the data management section and analysis, the results of the reviews are extracted and used in the synthesis, as in an umbrella review. This must be clarified prior to data extraction

*Thank you for this very helpful observation. We have now revised the second paragraph in the “Data extraction and management section” and specified that only information relevant to the processing of included primary studies will be extracted from the included systematic reviews:*

*“The following information will be extracted from the reviews: (a) review details (authors, year of publication); (b) search details (sources searched, range of included studies, number of studies included in the review); (c) details of quality appraisal (appraisal tool used and appraisal rating), if available.” (p. 15-16)*

 4. As a final point, I did note that the search terms did not include any synonyms for surrogate. I recommend 'substitute*' and 'alternate' to ensure the scope of this group.

*We appreciate this suggestion. As explained in response to Reviewer 1 (comment 5), our search strategy was informed by an information specialist and, at the time of receiving this feedback, searches had been completed. Due to the large workload and lack of new resources, the inclusion of new terms would not be feasible and we need to decline to conduct the search again. However, we will acknowledge the omission of the term as a study limitation in the report from this review.*

 5. There also appears a typographical error in the first paragraph of the Data extraction and management section where JBM is referred to. I believe it should be JBI.

*Thank you, this has now been corrected. (p. 14)*

 6. I wish you well with the review and look forward to reading the final outcomes which should have practical implications for the field.

*Thank you. We look forward to sharing the report from the study.*

# Peer review: Reviewer response for version 1

**DOI:** 10.21956/hrbopenres.14182.r27677

**Published:** 2020-08-10

**Authors:** Jessica E. Simon

**Affiliations:** 1 Department of Oncology, University of Calgary, Calgary, AB, Canada

**Is the rationale for, and objectives of, the study clearly described?** 

 This is an important systematic review addressing the issue of engagement in advance care planning (ACP) by community dwelling adults over 50 years of age. This is a key population who may benefit from participating in ACP with potential broader impacts on population health and heath system resource use. The theoretical framework selected for the analysis has the advantage of allowing the barriers and facilitators to engagement in ACP by community dwelling adults over 50 years of age to be understood in terms of behavioural change. The study should result in understanding how Capability, Motivation, and Opportunity at the individual, interpersonal, provider, and system levels of influence uptake of ACP by these adults.

 The paper provides an excellent background with a current consensus definition of ACP and relevant, succinct literature reviewed.

 One notable publication that was missing from the background section is Lovell and Yates (2014) ^1^. The authors might want to reconsider their sentence, *“..* *no study has synthesised available evidence to identify patterns across older adults, irrespective of their health status or condition, while taking stakeholder perspective and level of influence into account.*” The review above does explore the global contextual factors for ACP uptake that this protocol will be seeking to understand, although it does not use an organizing theory such as Michie’s COM-B model. Consider changing the sentence to, “…few studies have synthesized available evidence to identify patterns across older adults, irrespective of their health status or condition and these have used an organizing behaviour change theory and taken all stakeholder perspectives and level of influence into account.”

 One very minor grammatical edit could be considered. In a couple of places this phrase is used: “As readiness to ACP engagement…” . To increase ease of reading perhaps consider changing to “ As readiness for ACP engagement…” or, “As readiness to engage in ACP…”

**Is the study design appropriate for the research question?**

 The study design and search strategy is well described, using a hybrid systematic review with two phases. I am curious whether adding “public” as a setting to the search terms might be useful to identify additional references with healthy adults that the term “community” might not pick up. The authors do not comment specifically on whether “grey literature” references from the phase 1 systematic reviews will be included in phase 2 but these would seem an additional important source of information.

 Given the authors stated aim that, “The findings of this review may inform the development of an innovative and evidence-based interventions for ACP” I’m inclined to suggest considering an addition to your data extraction table to included whether the study evaluated an intervention strategy and what that intervention was and which COM-B factor it addressed may enable their analysis which seeks to “differentiate individual-level, service-level, and system-level factors that facilitate and/or impede stakeholders engagement in ACP” and assist the authors in moving into describing what intervention functions and policy categories have been published as the next step in applying Michie’s Behaviour Change Wheel.

**Are sufficient details of the methods provided to allow replication by others?**

 Yes, except that the timeframe proposed for the phase 1 systematic review search and any included/excluded languages are not provided. Please add this detail.

**Are the datasets clearly presented in a useable and accessible format? Yes the search terms are clearly presented and accessible.**

 Yes, this is well done.

Is the study design appropriate for the research question?

Yes

Is the rationale for, and objectives of, the study clearly described?

Yes

Are sufficient details of the methods provided to allow replication by others?

Yes

Are the datasets clearly presented in a useable and accessible format?

Yes

Reviewer Expertise:

Advance care planning and palliative care.

I confirm that I have read this submission and believe that I have an appropriate level of expertise to confirm that it is of an acceptable scientific standard.

## References

[1] : Advance Care Planning in palliative care: a systematic literature review of the contextual factors influencing its uptake 2008-2012. *Palliat Med* .2014;28(8) : 10.1177/0269216314531313 1026-35 10.1177/0269216314531313 24821708

# Response

**Published:** 2021-06-04

**Authors:** Monika Pilch

**Affiliations:** Trinity College Dublin, Ireland

We are grateful for the time taken by reviewers in providing a careful analysis of our protocol. We appreciate your positive comments and constructive suggestions to further improve our manuscript. We have revised the paper and have made a point-by-point reply to your comments, as follows:

 1. This is an important systematic review addressing the issue of engagement in advance care planning (ACP) by community dwelling adults over 50 years of age. This is a key population who may benefit from participating in ACP with potential broader impacts on population health and health system resource use. The theoretical framework selected for the analysis has the advantage of allowing the barriers and facilitators to engagement in ACP by community dwelling adults over 50 years of age to be understood in terms of behavioural change. The study should result in understanding how Capability, Motivation, and Opportunity at the individual, interpersonal, provider, and system levels of influence uptake of ACP by these adults.

*Thank you for these positive comments.*

 2. The paper provides an excellent background with a current consensus definition of ACP and relevant, succinct literature reviewed.

*Thank you for this positive comment.*

 3. One notable publication that was missing from the background section is Lovell and Yates (2014) ^1^ . The authors might want to reconsider their sentence, “..no study has synthesised available evidence to identify patterns across older adults, irrespective of their health status or condition, while taking stakeholder perspective and level of influence into account.” The review above does explore the global contextual factors for ACP uptake that this protocol will be seeking to understand, although it does not use an organizing theory such as Michie’s COM-B model. Consider changing the sentence to, “…few studies have synthesized available evidence to identify patterns across older adults, irrespective of their health status or condition and these have used an organizing behaviour change theory and taken all stakeholder perspectives and level of influence into account.”

*Thank you for this comment. We think the reviewer meant to state that these reviews have **not** used an organising behaviour change theory? To increase clarity, we applied the suggestion and changed the text in the relevant section (p. 6) to the following:*

*“Although several studies have been conducted to explore factors associated with the uptake of ACP among older adults in community settings; and these have been synthesised in previous systematic reviews (e.g.,* *De Vleminck et al., 2013* *;* *Ramsaroop et al., 2007* *;* *Risk et al., 2019* *;* *Solis et al., 2018* *), **few studies have** synthesised available evidence to identify patterns across older adults, irrespective of their health status or condition, while taking stakeholder perspective and level of influence into account. A synthesis utilising the BCW framework is necessary to identify influences within the contextual layers of health behaviour among older adults living in a community, while highlighting individual, interpersonal, provider, and system levels of influence.” *(p. 6)

 4. One very minor grammatical edit could be considered. In a couple of places this phrase is used: “As readiness to ACP engagement…” . To increase ease of reading perhaps consider changing to “ As readiness for ACP engagement…” or, “As readiness to engage in ACP…”

*Thank you, these changes have now been applied on pages 5 and 6.*

 5. The study design and search strategy is well described, using a hybrid systematic review with two phases. I am curious whether adding “public” as a setting to the search terms might be useful to identify additional references with healthy adults that the term “community” might not pick up.

*We appreciate this suggestion. Our search strategy was informed by an information specialist and, at the time of receiving this feedback, searches had been completed. Due to the large workload and lack of new resources, the inclusion of new terms would not be feasible and we need to decline to conduct the search again. However, we will acknowledge the omission of the term as a study limitation in the report from this review.*

 6. The authors do not comment specifically on whether “grey literature” references from the phase 1 systematic reviews will be included in phase 2 but these would seem an additional important source of information.

*Thank you for this comment. Prior to preparing this protocol, we completed a scoping search. The outcome of the scoping suggested a considerable amount of evidence available. Although we recognise the importance of “grey literature”, we also had to consider the limited scope of research, which is a part of a PhD project, and the need to complete it within the required timeframe. Therefore, we made a decision to exclude “grey literature” in our systematic review. This has now been explicitly stated in the text, by adding the following sentence:*

*“Although we acknowledge the importance of grey literature, due to envisaged amount of existing evidence on the topic, grey literature will not be considered in this review.”* *(p. 11)*

 7. Given the authors stated aim that, “The findings of this review may inform the development of an innovative and evidence-based interventions for ACP” I’m inclined to suggest considering an addition to your data extraction table to include whether the study evaluated an intervention strategy and what that intervention was and which COM-B factor it addressed may enable their analysis which seeks to “differentiate individual-level, service-level, and system-level factors that facilitate and/or impede stakeholders engagement in ACP” and assist the authors in moving into describing what intervention functions and policy categories have been published as the next step in applying Michie’s Behaviour Change Wheel.

*Thank you for this insightful comment. The aim of this review is to identify facilitators and barriers to ACP engagement, however, we appreciate potential benefits of including this additional information. We now specified that, if a study evaluated an intervention, we will include information about the name and  type of a strategy used. Additionally, where relevant information is available, we will identify specific COM-B factors associated with these interventions. We included this plan in the protocol and specified (in the data extraction sub-section) that “If a study evaluated an intervention, we will also extract the name of the strategy used. If relevant information is reported in a primary study, we will identify COM-B factors addressed in that intervention”.* (p. 15)

 8. Are sufficient details of the methods provided to allow replication by others?

 Yes, except that the timeframe proposed for the phase 1 systematic review search and any included/excluded languages are not provided. Please add this detail.

*We have now specified the timeframe for the phase 1 systematic review. Specifically, we added this underlined part to the sentence in the “Search Strategy section.*

*“* *A comprehensive search for systematic reviews will be conducted in each database from its inception (Phase 1) and this process will be completed in June 2020 ”. (p. 11)*

*With regards to the timeframe, w* *e plan to include only studies reported in English. This information was included on page 8 of the original protocol submission. “Only reviews and primary studies reported in English and published in peer-reviewed journals will be considered for inclusion.” (p. 8)*
